# Group 3 Innate Lymphoid Cells Protect Steatohepatitis From High-Fat Diet Induced Toxicity

**DOI:** 10.3389/fimmu.2021.648754

**Published:** 2021-03-15

**Authors:** Masahide Hamaguchi, Takuro Okamura, Takuya Fukuda, Kensuke Nishida, Yuta Yoshimura, Yoshitaka Hashimoto, Emi Ushigome, Naoko Nakanishi, Saori Majima, Mai Asano, Masahiro Yamazaki, Hiroshi Takakuwa, Masakazu Kita, Michiaki Fukui

**Affiliations:** ^1^Department of Endocrinology and Metabolism, Kyoto Prefectural University of Medicine, Kyoto, Japan; ^2^Agilent Technologies, Chromatography Mass Spectrometry Sales Department, Life Science and Applied Markets Group, Tokyo, Japan; ^3^Department of Immunology, Kyoto Prefectural University of Medicine, Kyoto, Japan

**Keywords:** ILC, IL-22, IL-23, NASH, NAFLD, steatosis

## Abstract

**Background and Aims:** Emerging evidence has revealed that innate lymphoid cells (ILCs) play a key role in regulating metabolic disorders. Here, we investigated the role of group 3 ILCs (ILC3s) in the modulation of Non-alcoholic fatty liver disease (NAFLD).

**Methods:** RORγ ^gfp/gfp^ (RORgt KI/KI) and Rag2^−/−^ mice with the administration of A213, RORgt antagonist, fed with a high-fat-diet (HFD) for 12 weeks, were used. We performed flow cytometry, real time PCR, and lipidomics analysis of serum and liver, and used RAW264.7 cells and murine primary hepatocytes *in vitro*.

**Results:** HFD increased ILC3s and M1 macrophages in the liver, and RORgt KI/KI mice deficient in ILC3 showed significant fatty liver, liver fibrosis and significantly increased palmitic acid levels in serum and liver. In addition, administration of A213 to Rag2^−/−^ mice caused significant fatty liver, liver fibrosis, and a significant increase in serum and liver palmitate concentrations, as in RORgt KI/KI mice. Addition of palmitc acid stimulated IL-23 production in cell experiments using RAW264.7. IL-22 produced by ILC3s inhibited the palmitate-induced apoptosis of primary hepatocytes.

**Conclusions:** HFD stimulates IL-23 production by M1 macrophages, thus promoting ILC3 proliferation, whereas IL-22 secreted by ILC3s contributes to the upregulation of hepatic lipid metabolism and has anti-apoptosis activity.

**Graphical Abstract:**
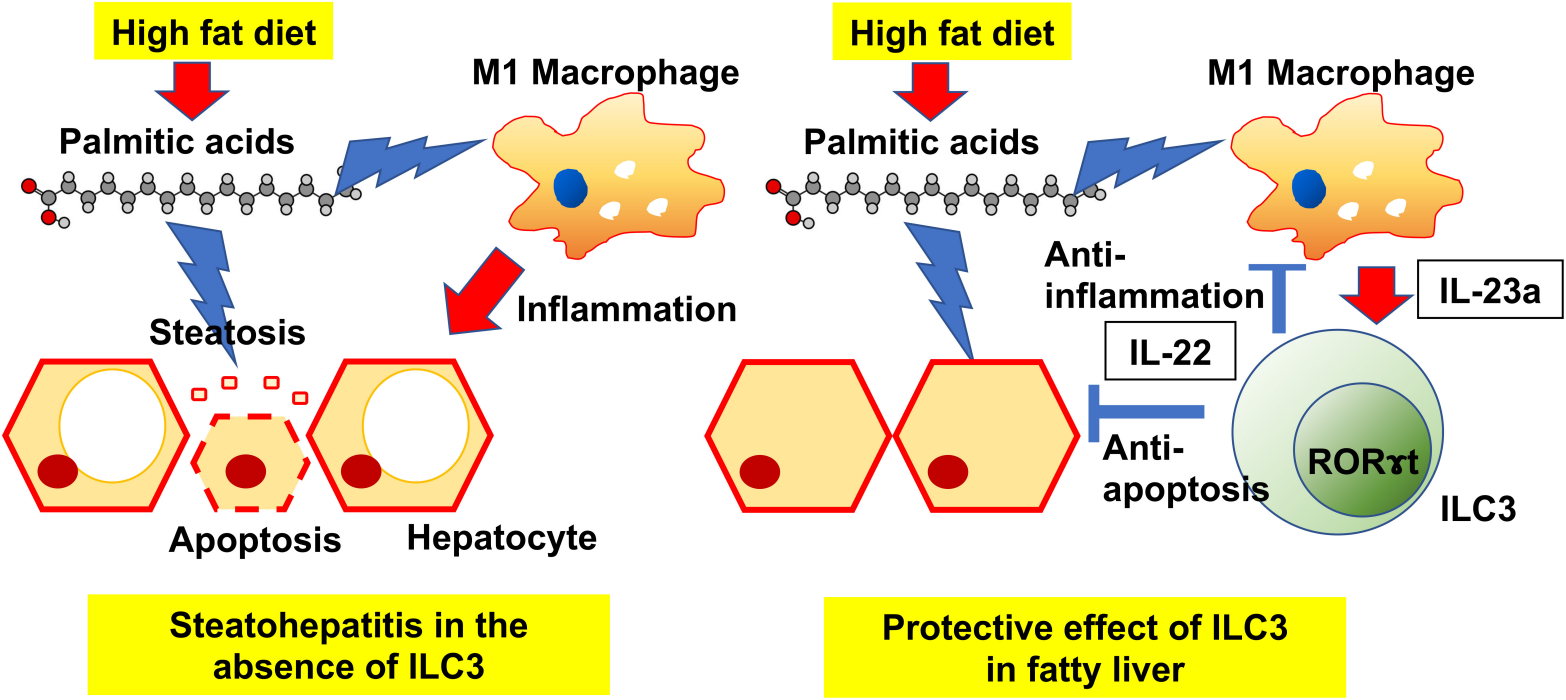


## Introduction

Non-alcoholic steatohepatitis (NAFLD) is the leading cause of chronic liver disease based on metabolic disturbances caused by overeating and lack of exercise, and encompasses histopathological features ranging from simple steatosis to non-alcoholic steatohepatitis (NASH) (1–3). The histological severity of NAFLD is correlated with liver-related morbidity and mortality (3), and knowing the mechanisms underlying the progression of NAFLD is important for devising treatment strategies. Although the detailed mechanism of immunomodulation in the liver is unknown, the cytokine production regulated by the activation of the immune system has a pivotal role in the progression of NAFLD (4).

Innate immunity has been reported to be a relevant in NAFLD (5, 6). Innate lymphoid cells (ILCs) lack antigen-specific receptors and therefore, do not require recombination activating gene (RAG) proteins for development (7). Nevertheless, ILCs respond to cytokines produced by surrounding macrophages, dendritic cells, and epithelial cells (8, 9). It is known that three lineages of ILCs exist (group 1 ILCs, group 2 ILCs, and group 3 ILCs) (9). ILC3s express RAR-related orphan receptor-gamma t (RORγt) (10), IL-23 receptor (11), and product IL-22 after stimulation by IL-23 (12). The proportion of hepatic ILC3 is rare, but it has been shown to be involved in protection and pathogenesis via secretion of cytokines (such as IL-22 and IL-17) in several liver diseases (13). The role of IL-22 in liver disease has been widely studied (14), and since the major cellular source of IL-22 in the liver is ILC3, ILC3 may play an important role in liver disease. Additionally, M1 macrophages secrete an abundance of proinflammatory cytokines and adipokines, which induce chronic inflammation (15). On the other hand, a certain number of macrophages are rather rich in anti-inflammatory cytokines such as IL-10, which are called M2 macrophages. These immune cells, along with ILCs, are involved in innate immunity and have been reported to be involved in various lifestyle diseases, including NAFLD, by regulating chronic inflammation.

In the present study, we focused on the role of type 3 innate lymphoid cells (ILC3s) and M1/M2 macrophages in the modulation of NAFLD and the potential therapeutic targets using RORγt ^gfp/gfp^ mice and Rag2^−/−^ mice with the administration of A213, which is RORγt antagonist, with a high fat diet. Moreover, palmitic acid-induced lipotoxicity has been reported to play a key role in the pathogenesis of NAFLD (2). Therefore, the association between palmitic acid and innate immunity was also investigated.

## Materials and Methods

### Animals

RORc(γt)-EGFP mice were obtained from the Jackson Laboratory (007572) and are named RORγt ^gfp/gfp^ mice (RORgt KI/KI mice) in the manuscript. Heterozygous mice, RORγt ^gfp/wt^ mice (RORgt KI/w mice) were used as a control group. B6(Cg)-Rag2tm1.1Cgn/J (Rag2^−/−^) mice were obtained from the Jackson Laboratory (008449). As for the high-fat diet (HFD), we used HFD60, which contained 60 cal% fat (lard), 20 cal% carbohydrate, and 20 cal% protein (Oriental Yeast Co., Tokyo).

Mice were fed a normal diet (ND; 344.9 kcal/100 g, fat kcal 4.6 %; CLEA Japan, Tokyo) or HFD from 8 to 20 weeks of age. The animal experiments in this study were performed in accordance with the guidelines and regulations for the Care and Use of Laboratory Animals. The protocols were approved by the Institutional Animal Ethics Committee of the Kyoto prefectural University (Kyoto, Japan) (M2019-41).

Moreover, we tried to create RORgt KI/KI Rag2^−/−^ double knockout (DKO) mice, however, the DKO mice seemed to die *in utero* and we could not produce them. Therefore, to reproduce the DKO mice, we administered the RORgt antagonist A213 to Rag2KO mice to knock out ILC3 (16). A213 was orally administered to Rag2^−/−^ mice at 18 weeks of age three times for three consecutive days, and they were sacrificed after 2 weeks at 20 weeks of age.

### Analytic Procedures and Glucose Tolerance Tests

To measure weight, mice were fasted overnight (~14–16 h), and weights were measured once a week. In 20-week-old mice, an intraperitoneal glucose tolerance test (iPGTT) (2 g/kg of body weight) was performed after 16-h fasts, and blood glucose was measured by collecting a drop of blood, using a glucometer at the times indicated (Gultest Neo Alpha; Sanwa Kagaku Kenkyusho, Nagoya, Japan). The area under the curve (AUC) of the iPGTT result was analyzed.

### Biochemistry

In designated experiments, mice were fed the HFD from 8 to 20 weeks of age. Mice were fasted for 3 h and sacrificed. Blood samples were taken from fasted mice and alanine aminotransferase (ALT) levels, total cholesterol, triglycerides, and non-esterified fatty acids (NEFA) were measured. The biochemical examinations were performed at FUJIFILM Wako Pure 18 Chemical Corporation (Osaka, Japan).

### Measurement of Fatty Acids in the Liver

Using gas chromatography-mass spectrometry (GC/MS), Agilient 7890B/5977B (Agilient Technologies, Santa Clara, CA, USA), liver and serum palmitic acid compositions were measured. Briefly, fifteen μg of liver and 25 μL of serum were methylated with a fatty acid methylation kit (nacalai tesque, Kyoto, Japan), and products were added to a Varian capillary column (DB-FATWAX UI; Agilent Technologies). Using CP-Sil 88 for FAME (100 m × an inner diameter of 0.25 mm × membrane thickness of 0.20 μm, Agilent Technologies) separation of fatty acid was performed; during this operation, the column temperature was maintained at 100°C for 4 min and then increased step by step by 3°C/min to 240°C and held there for 7 min. Then, the sample was shoot with split ratio 5:1 in split mode and each fatty acid methyl ester was observed in selected ion monitoring mode. Normalization was performed with the peak height of the C17:0 internal standard.

### Liver Histology

Obtained liver was fixed with 10% buffered formaldehyde and embedded in paraffin, and then stained with hematoxylin and eosin, Masson's trichrome, and Sirius red stain. Using BZ-X710 fluorescence microscope (Keyence, Osaka, Japan), images were obtained. To evaluate NAFLD severity, the NAFLD activity score (NAS) (17), which is a well-known standard, used for assessing NASH severity and measuring changes in NAFLD, were checked. NAS was evaluated by a trained hepatopathologist, with masking the experimental conditions (17). Briefly, the scoring system consisted with 14 histological features, four of which were evaluated semi-quantitatively: hepatocellular ballooning (0–2), lobular inflammation (0–2), steatosis (0–3), and fibrosis (0–4). In addition, to assess the fibrosis, Stage 1 was classified as 1A for mild peri-central perisinusoidal fibrosis, 1B for moderate or greater perisinusoidal fibrosis, and 1C for fibrosis in the portal region or peri-portal vein. Stage 2 was classified for perisinusoidal and periportal fibrosis, Stage 3 was classified for bridging fibrosis, and Stage 4 was classified for liver cirrhosis.

### Protocols for Isolation of Mononuclear Cells From Liver

A needle was inserted into the portal vein with opening the abdominal cavity, under deep anesthesia. After perfusion with 20 mL of pH 7.0 phosphate buffered salts (PBS), the liver was removed. Procedure of hepatic lymphocytes isolation were follows. The obtained liver was filtrated with a 200-gauge stainless steel mesh filter, and then suspended with Roswell Park Memorial Institute (RPMI) 1640 medium, containing 20 mL/L fetal bovine serum (FBS, 2%). Then, suspension of liver cell was performed with centrifugation at 1,500 rpm. The pellet was resuspended with 40% Percoll solution, overlaid on an equal volume layer of 60% Percoll solution, and then suspension was performed with centrifugation at 2,000 rpm for 20 min at room temperature. Lastly, the cells were aspirated from the Percoll interface (buffy coat), then pellet was obtained after centrifugation, and washed twice with PBS with 2% FCS before use (18).

### Tissue Preparation and Flow Cytometry

FACS Canto II and FlowJo version 10 software (TreeStar, Ashland, OR, USA) were used for obtained data and analyzation. Gating of innate lymphoid cells was performed with following strategies: Biotin-CD3e (100304; clone: 145-2C11; 1/200; eBioscience, San Diego, CA, USA), Biotin- CD45R/B220 (103204; clone: RA3–6B2; 1/200; eBioscience), Biotin-Gr-1 (108404; clone: RB6-8C5; 1/200; eBioscience), Biotin-CD11c (117304; clone: N418; 1/200; eBioscience), Biotin-CD11b (101204; clone: M1/70; 1/200; eBioscience), Biotin-Ter119 (116204; clone: TER-119; 1/200; eBioscience), Biotin-FceRIa (134304; clone: MAR-1; 1/200; eBioscience), FITC-Streptavidin (405202; 1/500; eBioscience), PE-Cy7-CD127 (135014; clone: A7R34; 1/100; eBioscience), Pacific Blue-CD45 (103116; clone: 30-F11; 1/100; eBioscience), PE -GATA-3 (clone TWAJ, 1/50; eBioscience), APC -RORγ (clone AFKJS-9, 1/50, eBioscience), and Fixable Viability Dye eFluor 780 (1/400; eBioscience) (19, 20) (Supplementary Figure 1). ILC3s were gated for CD45+ Live & Dead- Lin- CD127+ RORg+ GATA-3-. Additionally, M1 and M2 macrophages was evaluated by the following strategies: APC- CD45.2 (17045482; clone: 104, 1/50; eBioscience), PE-F4/80 (12480182; clone: BM8, 1/50, eBioscience), APC-Cy7-CD11b (47011282; clone: M1/70, 1/50; eBioscience), FITC-CD206 (MA516870; clone: MR5D3, 1/50, eBioscience), and PE-Cy7-CD11c (25011482; clone: N418, 1/50, eBioscience) (21) (Supplementary Figure 2). M1 macrophages were gated for CD45+ F4/80+ CD206- CD11c+ and M2 macrophages were gated for CD45+ F4/80+ CD206+ CD11c-.

### Isolation and Culture of Murine Primary Hepatocytes

Murine primary hepatocytes were isolated and cultured using the following protocol (15). C57/BL6J male mice aged 8–10 weeks were used. Reagents for hepatocyte isolation and culture were pre-warmed in Solution 1: Hank's Balanced Salt Solution (HBSS, Wako), EDTA 0.5 mM, pH = 8, and Solution 2: Dulbecco's Modified Eagle Medium (DMEM, Wako) 40 ml and Collagenase Type I 32 mg (0.8 mg/mL, Worthington Biochemical). While the heart is still beating, the catheter (22G feeding needle/round tip, connected to Solution 1) was inserted into the portal vein and inferior vena cava, and kept in place by applying a surgical knot. Solution 1 was manually injected into the portal vein for 5–7 min (flow rate 5 mL/min), and Solution 2 was similarly injected for 5–7 min. Then, liver was collected in a tube containing 5 mL of DMEM on ice. The liver was cut and passed through a cell strainer. Cells were centrifuged at 50 g for 1 min. The pellet was suspended in primary hepatocyte culture medium (Williams' E Medium, supplemented with 5% FCS, 2 mM glutamine, 0.1 μg/ml glucagon, 10 μg/ml insulin, 0.7 μg/ml dexamethasone, and 1% penicillin/streptomycin).

### Murine Macrophage Cell Culture and Flow Cytometry

RAW264.7 cells (the cell line RAW264.7, KAC Co., Ltd., Kyoto, Japan) was used for evaluation of murine macrophage. RAW264.7 cells were seeded into 24-well plates and grown in DMEM with 10% FBS. After adding the ethanol, 50, 100, and 200 μM palmitic acid for 24 h, RAW264.7 cells were pre-treated with phorbol myristiric acid (PMA) at the indicated concentrations for 20 min prior to stimulation with 1 μM ionomycin for cytokine release.

FACS Canto II and FlowJo version 10 software were used for obtained data and analyzation. *Il-23* positive cells were evaluated by the following strategies: Pacific Blue-CD45 (103116; clone: 30-F11; 1/100; eBioscience), PE-F4/80 (12480182; clone: BM8, 1/50, eBioscience), and FITC-IL23 (53702382; clone: fc23cpg; 1/50, eBioscience) (22).

### Caspase-3 Antibody Staining Protocol

Primary hepatocytes were cultured in 8-well chamber slides and immunocytochemistry was performed on them. Primary hepatocytes were fixed in 4 % paraformaldehyde and incubated with primary monoclonal antibodies: Anti-Cleaved Caspase-3 antibody (ab32042, abcam, Cambridge, UK), diluted in PBS/1%, BSA/0.3%, Triton^TM^ X-100 (Sigma-Aldrich, St. Louis, MO, USA) overnight at 4°C, and a Texas-red-conjugated anti-mouse secondary antibody (Jackson ImmunoResearch) diluted in PBS/1%, BSA/0.3%, Triton^TM^ X-100 overnight at 4°C for 1 h. Nuclei were stained with DAPI (Sigma-Aldrich). Images were captured with the BZ-X710 fluorescence microscope, and the ratio of Caspase 3-positive cells per image was analyzed using ImageJ (NIH).

### Cotreatment With Palmitic Acids and IL-22 Into Primary Hepatocytes

Primary hepatocytes were treated with ethanol, 200 μM of palmitic acid or 200 μM of palmitic acid, and 10 ng/ml of IL-22 for 24 h.

### Quantitative RT–PCR

Total RNA was extracted from the liver after perfusion with PBS and preserved in RNA (Ambion, Austin, TX, USA) until the extraction of RNA. Extraction of RNA was performed by the TRIzol reagent (Life Technologies, Carlsbad, CA) or RNeasy Micro Kit (Qiagen, Hilden, Germany), according to the manufacturer's procedure. Synthetization of the first strand of complementary DNA was performed by the High capacity RT-PCR Kit (Applied Biosystems, Carlsbad, CA). Quantitative polymerase chain reaction (qPCR) was performed to measure levels of mRNA using TaqMan gene expression kit (Applied Biosystems). A VIC®-labeled probe for *Gapdh* was used for normalization.

### Statistics

The differences between two groups were assessed by *t*-test for parametric continuous values or Mann–Whitney *U*-test for non-parametric continuous values. The differences in categorized variables between the two groups was assessed by Pearson's chi-square test. The differences in continuous variables among more than three groups were assessed by One-way analysis of variance (ANOVA) test with Turkey's honestly significant difference for multiple comparisons or the Kruskal-Wallis test with Steel-Dwas for multiple comparisons, respectively. We used Prism version 8.0 software (GraphPad, San Diego, CA). *P*-values of less than 0.05 were considered significant.

## Results

### Deficiency of ILC3s Led to Liver Fibrosis, Impaired Glucose Tolerance, and Dyslipidemia

**Figure 1:**
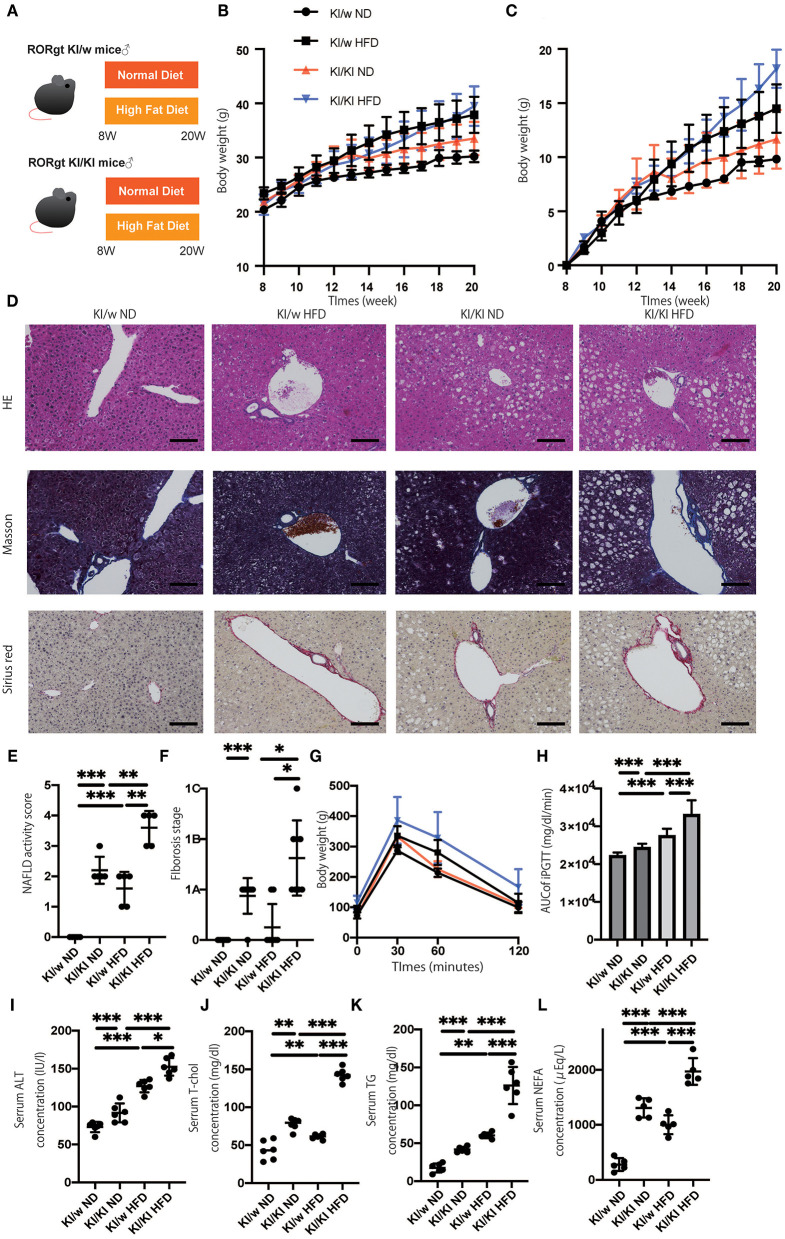
The deficiency of RORgt induced a significant fatty liver and fibrosis. **(A)** Eight-week-old RORgt GFP KI/w mice and RORgt GFP KI/KI mice were fed with normal diet (ND) or high fat diet (HFD) for 12 weeks. When the mice reached 20 weeks of age, the mice were sacrificed. **(B)** Body weight changes (*n* = 12) are shown. RORgt KI/KI mice fed with ND showed significant increased body weight compared to RORgt KI/w mice fed with ND (*p* = 0.013). **(C)** Weight gain (*n* = 12) are shown. Weight gain in the ND group was significantly higher in KI/w mice than in KI/KI mice from 12 weeks of age in the ND group and from 17 weeks of age in the HFD group. **(D)** Representative images of Hematoxylin & Eosin and Masson trichrome, and Sirius red stained liver sections. Liver tissues were collected, after measuring body weight at 20 weeks of age. The scale bar shows 100 μm. Lipolysis was significantly exacerbated in KI/KI mice. **(E,F)** NAFLD activity scores and fibrosis stage of NAFLD activity score (*n* = 5). RORgt KI/KI mice significantly increased both hepatic fat accumulation and fibrosis stage compared with RORgt KI/w mice both fed with ND and HFD (ND: *p* < 0.001, HFD: *p* < 0.001). Data are mean ± SEM; *p < 0.05, **p < 0.01, ***p < 0.001 by one-way ANOVA. **(G,H)** The results of iPGTT. When the mice reached 20 weeks of age, an iPGTT (2 g/kg body weight) was performed and the data's AUC was analyzed (*n* = 12). The RORgt KI/KI mice showed significant impaired glucose tolerance in both feeding with ND and HFD (ND: *p* < 0.001, HFD: *p* < 0.001). **(I–L)** Biochemical examinations, alanine aminotransferase (ALT), total cholesterol (T-Chol), triglycerides (TG) and non-esterified fatty acid (NEFA), were performed (*n* = 12). (ALT: ND, *p* < 0.001; HFD, *p* = 0.034. T-chol: ND, *p* = 0.004; HFD, *p* < 0.001. TG: ND, *p* < 0.001; HFD, *p* < 0.001. NEFA: ND, *p* < 0.001; HFD, *p* < 0.001). Data are mean ± SEM; *p < 0.05, **p < 0.01, ***p < 0.001 by one-way ANOVA.

To assess the function of ILC3s, which increased in fatty liver, RORgt KI/w mice were compared to RORgt KI/KI mice (Figure 1A). The functions of ILC3 are impaired in RORgt KI/KI mice (12, 23). RORgt KI/KI mice fed with ND showed significantly increased body weight, compared to RORgt KI/w mice fed with ND (*p* = 0.013) (Figure 1B). Additionally, weight gain in the ND group was significantly higher in KI/w mice than in KI/KI mice from 12 weeks of age in the ND group and from 17 weeks of age in the HFD group (Figure 1C). Hepatic fat accumulation of RORgt KI/KI mice was higher than that of RORgt KI/w mice (ND: *p* < 0.001, HFD: *p* < 0.001) (Figures 1D,E). In addition, liver fibrosis of RORgt KI/KI mice fed with HFD was worse than that of the others (Figures 1D,F). In both groups fed with ND or HFD, RORgt KI/KI mice showed impaired glucose tolerance (ND: *p* < 0.001, HFD: *p* < 0.001), compared with RORgt KI/w (Figures 1G,H). In addition, ALT, total cholesterol, triglycerides, and NEFA in RORgt KI/KI mice were also higher than those of the others (Figures 1I–L). Taken together, deficiency of ILC3s and a high fat diet led to liver fibrosis, impaired glucose tolerance, and dyslipidemia.

### ILC3s and M1 Macrophages Increased in Fatty Liver of Mice Fed With High Fat Diet

**Figure 2:**
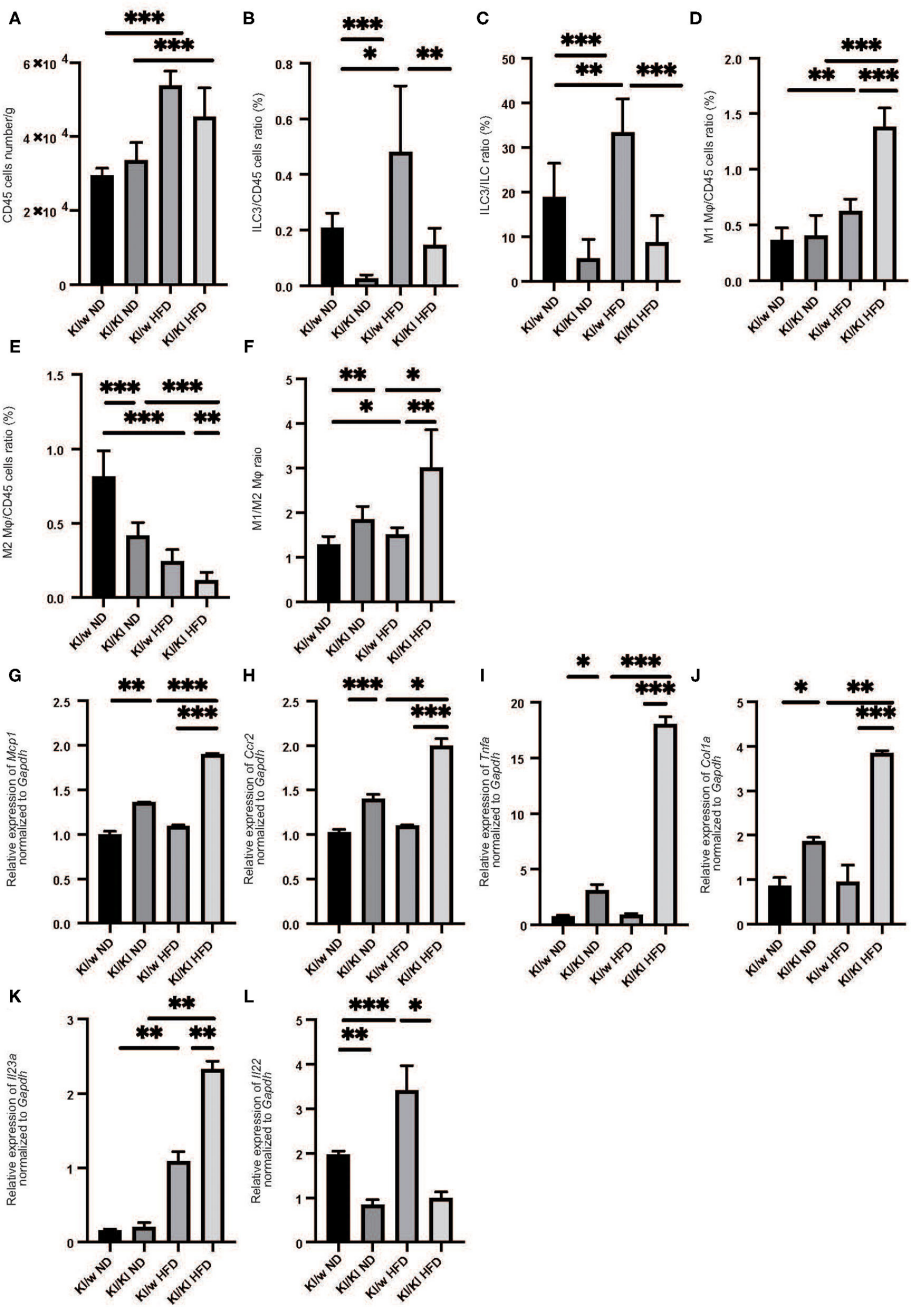
The deficiency of RORgt induced decreasing of ILC3s, and increasing M1 macrophages and decreasing M2 macrophages. **(A)** The number of CD45 positive cells in liver (/g). **(B,C)** The ratio of ILC3s in CD45 positive cells. The ratio of liver ILC3s in CD45 positive cells in RORgt KI/KI mice significantly decreased, compared to that in RORgt KI/W mice (*p* < 0.001). Both in RORgt KI/W and RORgt KI/KI mice, the number of ILC3s significantly increased by feeding with HFD (RORgt KI/W: *p* < 0.001, RORgt KI/KI: *p* < 0.001). **(D)** The ratio of M1 macrophages in CD45 positive cells in liver. The ratio of M1 macrophages in CD45 positive cells in RORgt KI/KI mice fed with HFD was significantly higher than that in RORgt KI/W mice fed with HFD (*p* < 0.001). **(E)** The ratio of M2 macrophages in CD45 positive cells in liver. The ratio of M2 macrophages in CD45 positive cells in RORgt KI/KI mice fed both with ND and HFD was significantly lower than that in RORgt KI/W mice (ND: *p* < 0.001; HFD: *p* = 0.006). **(F)** The ratio of M1 macrophages to M2 macrophages (M1/M2) in liver. The ratio of M1/M2 macrophages in RORgt KI/KI mice fed both with ND and HFD was significantly higher than that in RORgt KI/W mice (ND: *p* = 0.032; HFD: *p* = 0.002). Data are mean ± SEM; *p < 0.05, **p < 0.01, ***p < 0.001 by one-way ANOVA. **(G–K)** The relative expression of *mRNA Mcp1, Ccr2, Tnf*α*, Col1a*, and *Il23a* of indicated genes in liver normalized to *Gapdh* (*n* = 6). The expression of *Mcp1, Ccr2, Tnf*α, and *Col1a* in RORgt KI/KI mice was significantly higher than that in RORgt KI/W mice both in group fed with ND and HFD (*Mcp1*: ND; *p* < 0.001, HFD; *p* < 0.001. *Ccr2*: ND; *p* < 0.001, HFD; *p* < 0.001. *Tnf*α: ND; *p* = 0.024, HFD; *p* < 0.001. *Col1a*: ND; *p* = 0.019, HFD; *p* < 0.001) **(G–J)**. The expression of *Il-23a* in liver in RORgt KI/KI mice fed a HFD was significantly higher than that in RORgt KI/W mice fed a HFD (*p* = 0.009) **(K)**. The expression of *Il-22* in liver in RORgt KI/KI mice fed a HFD was significantly lower than that in RORgt KI/W mice fed a HFD (*p* < 0.001) **(L)**. Data are mean ± SEM; *p < 0.05, **p < 0.01, ***p < 0.001 by one-way ANOVA.

We next examined the changes of ILCs and macrophages in the liver by flow cytometry (Supplementary Figures 1, 2). The number of CD45-positive cells in fatty liver was significantly increased in HFD fed RORgt KI/w mice (*p* < 0.001) (Figure 2A). In addition, the number of ILC3s in liver per CD45 positive cells significantly increased due to HFD (*p* < 0.001) (Figures 2B,C).

We also investigated the changes in M1/M2 macrophages in the liver (Supplementary Figure 2). M1 macrophages in the liver of RORgt KI/w mice fed with HFD were significantly higher than that in RORgt KI/w mice fed with ND (*p* < 0.001) (Figure 2D), whereas M2 macrophages in the fatty liver of RORgt KI/w mice fed with HFD were significantly lower than that of RORgt KI/w mice fed with ND (*p* < 0.001) (Figure 2E). Moreover, the M1/M2 macrophage ratio in the liver of RORgt KI/w mice fed with HFD was significantly higher than that in the liver of RORgt KI/w fed with ND (*p* = 0.044) (Figure 2F).

### Deficiency of ILC3s Increased M1 Macrophages and Decreased M2 Macrophages

Feeding a high fat diet increased ILC3s in liver, whereas ILC3s significantly decreased in liver of RORgt KI/KI mice (Figures 2B,C). Moreover, M1 macrophages in the fatty liver of RORgt KI/KI mice was significantly higher than that of RORgt KI/w mice, and M2 macrophages in the fatty liver of RORgt KI/KI mice was significantly lower than that of RORgt KI/w mice (Figures 2D–F).

### Deficiency of ILC3s Led to the Increased Expression of Genes Related With Inflammation and Il-23a in Liver

The expression of *Mcp1, Ccr2*, and *Tnfa* in the liver of RORgt KI/w mice fed with HFD (*Mcp1*: 1.37, *Ccr2*: 1.41, *Tnfa*: 0.92) tended to be higher than those with ND, however, they were not statistically significant (*Mcp1*: 1.00, *p* = 0.057, *Ccr2*: 1.02, *p* = 0.093, *Tnfa*: 0.77, *p* = 0.063). On the other hand, the expression of *Mcp1, Ccr2, Tnf*α, and *Col1a* in RORgt KI/KI mice was significantly higher than that in RORgt KI/w mice both in mice fed with ND and in mice fed with HFD (Figures 2G-J). In addition, the expression of *Il23a* in the liver of RORgt KI/KI mice fed with HFD was significantly higher than that of the others (Figure 2K), whereas the expression of *Il-22* in liver in RORgt KI/KI mice fed a HFD was significantly lower than that in RORgt KI/W mice fed a HFD (*p* < 0.001) (Figure 2L).

### Palmitic Acids Significantly Increased in Both Serum and Liver of RORgt KI/KI Mice

**Figure 3:**
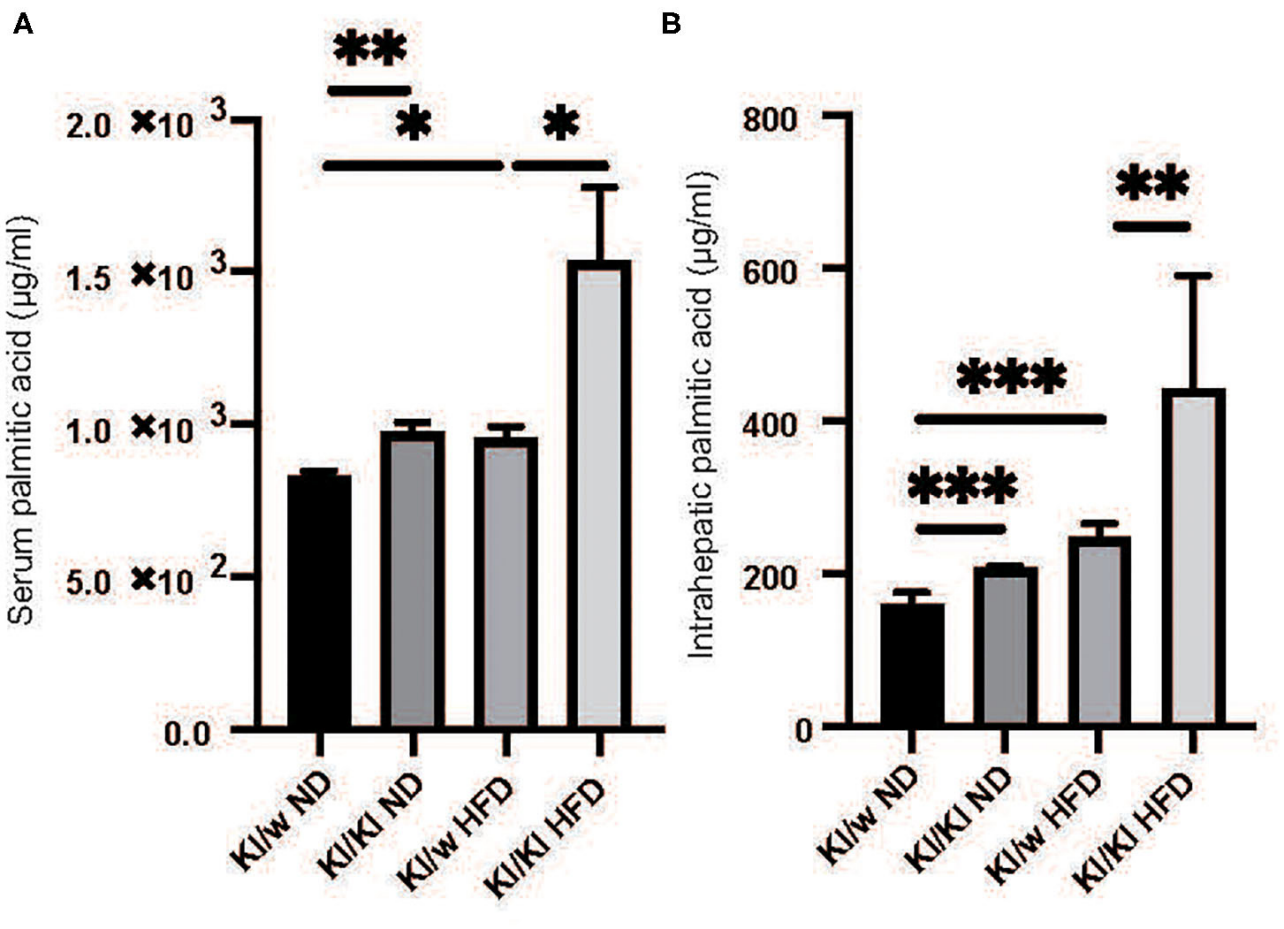
The deficiency of RORgt caused increasing of palmitic acid. **(A)** Serum palmitic acid (μg/ml). **(B)** Intrahepatic palmitic acid (μg/mg). Serum and intrahepatic palmitic acids of RORgt KI/KI mice were significantly higher than those of RORgt KI/W mice fed with both normal diet (ND) and high fat diet (HFD) (Serum: ND, *p* = 0.025; HFD, *p* < 0.001. Liver: ND, *p* < 0.001; HFD, *p* = 0.009). Data are mean ± SEM; *p < 0.05, **p < 0.01, ***p < 0.001 by one-way ANOVA.

Fatty acids in both serum and liver were analyzed by the GC-MS system. Serum and intrahepatic palmitic acids of RORgt KI/KI mice were significantly higher than those of RORgt KI/w mice fed with both diets (Serum: ND, *p* = 0.025; HFD, *p* < 0.001. Liver: ND, *p* < 0.001; HFD, *p* = 0.009) (Figures 3A–D).

### Rag2^–/–^ Mice Fed With High Fat Diet Showed Fat Accumulation in Liver, but Not Liver Fibrosis

**Figure 4:**
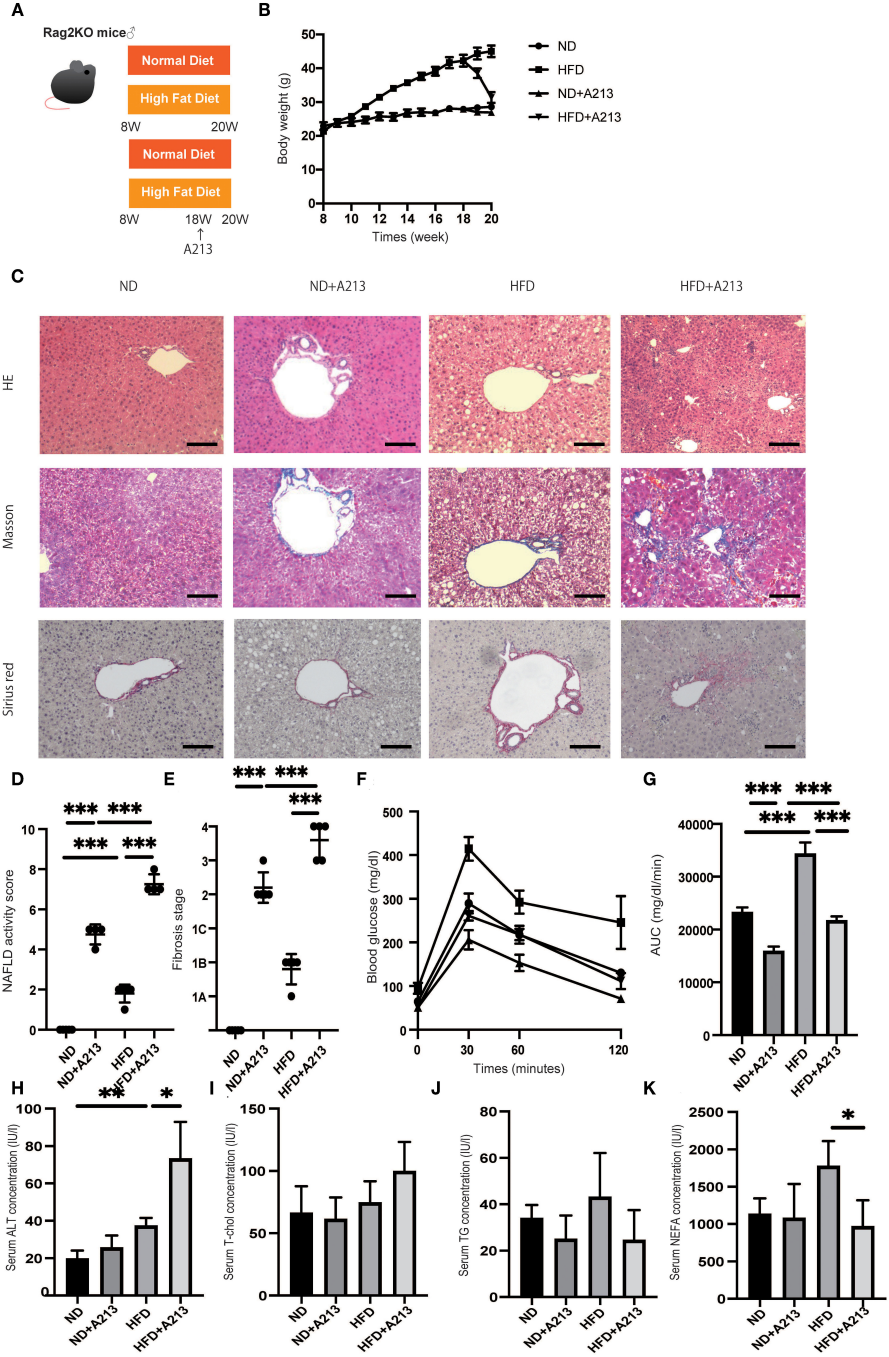
Rag2^−/−^ mice fed with high fat diet did not show liver fibrosis, whereas the administration of A213, RORgt antagonist, significantly aggravated liver fibrosis. **(A)** Eight-week-old Rag2^−/−^ mice were fed with normal diet (ND) or high fat diet (HFD) for 12 weeks. In addition, eighteen-week-old Rag2^−/−^mice were administered A213 orally three times for three consecutive days. When the mice reached 20 weeks of age, the mice were sacrificed. **(B)** Body weight changes (*n* = 6) are shown. **(C)** Representative images of Hematoxylin & Eosin (HE), Masson trichrome, and Sirius red stained liver sections. Liver tissues were collected, after measuring body weight at 20 weeks of age. The scale bar shows 100 μm. **(D,E)** NAFLD activity scores and fibrosis stage (*n* = 6). NAFLD activity score of mice with HFD was higher than that of mice with ND (*p* < 0.001). On the other hand, fibrosis stage score was not different between groups. Moreover, the administration of A213 aggravated NAFLD activity scores and fibrosis in both groups fed with ND and HFD. **(F,G)** The results of iPGTT. When the mice reached 20 weeks of age, an iPGTT (2 g/kg body weight) was performed and the data's AUC was analyzed (*n* = 6). Blood glucose levels of mice fed with HFD were higher than those of mice fed with ND. On the other hand, the administration of A213 did not exacerbate impaired glucose tolerance. **(H-K)** Biochemical examinations, alanine aminotransferase (ALT), total cholesterol (T-Chol), triglycerides (TG) and non-esterified fatty acid (NEFA), were performed (*n* = 6) (ALT: ND, *p* = 0.202; HFD, *p* = 0.036. T-chol: ND, *p* = 0.695; HFD, *p* = 0.069. TG: ND, *p* = 0.323; HFD, *p* = 0.060. NEFA: ND, *p* = 0.086; HFD, *p* = 0.017).

To confirm the increase in ILC3s in the fatty liver and the association between ILC3 and macrophages, we aimed to eliminate α/β-TCR T cells, γ/δ-TCR T cells, and B cells from the mice. For this purpose, we used Rag2^−/−^ (α/β-TCR T cell^−^, γ/δ-TCR T cell^−^, B cell^−^) mice with HFD for 12 weeks, and A213, RORgt antagonist, was orally administered to them to deplete ILC3 (Figure 4A). The body weight of Rag2^−/−^ mice fed with HFD was significantly higher than that of Rag2^−/−^ mice fed with ND (*p* < 0.001), whereas A213 administration decreased the body weight of both group fed with ND and HFD (ND: *p* = 0.016, HFD: *p* < 0.001) (Figure 4B). Representative liver histology of Rag2^−/−^ mice is shown in Figure 4C. Rag2^−/−^ mice fed with HFD showed significant fat accumulation in the liver, together with an early stage of fibrosis (*p* < 0.001) (Figures 4D,E).

Surprisingly, A213 administration caused an increase in intrahepatic fat accumulation and worsening of hepatic fibrosis in both groups fed with ND and HFD. Additionally, Rag2^−/−^ mice fed with HFD showed significantly impaired glucose tolerance (*p* < 0.001) (Figures 4F,G). On the other hand, despite worsening fatty liver and liver fibrosis, impaired glucose tolerance was mild in the A213 group. In the biochemical examinations, ALT levels were significantly increased by A213 administration in the HFD group; total cholesterol levels tended to increase in the HFD group. Serum triglycerides levels of mice fed with HFD tended to be higher than those with ND, however, it was not statistically significant (ND: 34 ± 5.0 mg/dl, HFD: 52.5 ± 15.3 mg/dl, p = 0.191). NEFA was significantly decreased by A213 administration (Figures 4H–K).

**Figure 5:**
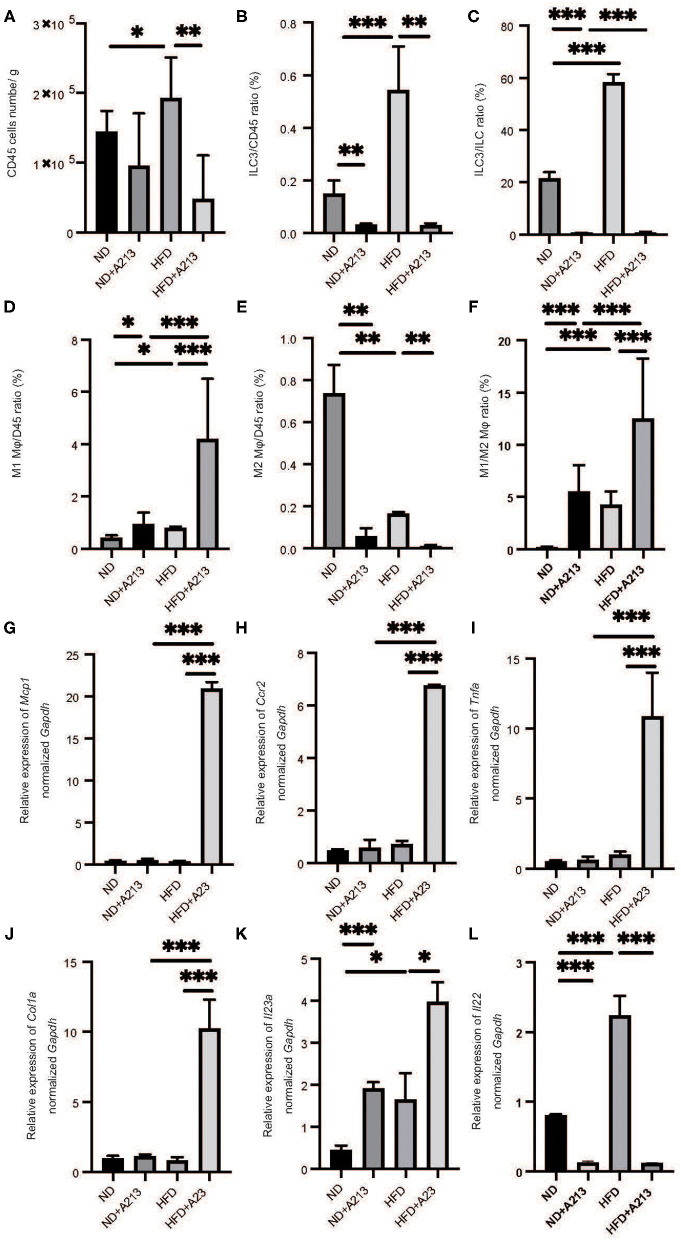
The administration of A213 induced decreasing of ILC3s, and increasing M1 macrophages and decreasing M2 macrophages. **(A)** The number of CD45 positive cells in liver (/g) (*n* = 6). **(B)** The ratio of ILC3s in CD45 positive cells (*n* = 6). **(C)** The ratio of ILC3s in ILCs. The number of CD45 positive cells and ILC3 of mice with HFD were higher than those of mice with ND (*p* < 0.001). On the other hand, the administration of A213 significantly decreased ILC3s. **(D)** The ratio of M1 macrophages in CD45 positive cells in liver. The ratio of M1 macrophages in CD45 positive cells in liver in Rag2^−/−^ mice fed with HFD was significantly higher than that in Rag2^−/−^ mice fed with ND (*p* = 0.023). The administration of A213 significantly increased the ratio of M1 macrophages in both groups fed with ND and HFD (ND: *p* = 0.024, HFD: *p* < 0.001). **(E)** The ratio of M2 macrophages in CD45 positive cells in liver. The ratio of M2 macrophages in CD45 positive cells in liver in Rag2^−/−^ mice fed with HFD was significantly lower than that in Rag2^−/−^ mice fed with ND (*p* = 0.019). The administration of A213 significantly decreased the ratio of M2 macrophages in both groups fed with ND and HFD (ND: *p* = 0.005, HFD: *p* = 0.002). **(F)** The ratio of M1 macrophages to M2 macrophages (M1/M2) in liver. M1/M2 macrophages ratio in liver in Rag2^−/−^ mice fed with HFD was significantly higher than that in Rag2^−/−^ mice fed with ND (*p* = 0.002). The administration of A213 significantly decreasedM1/M2 macrophages ratio in both groups fed with ND and HFD (*p* < 0.001). Data are mean ± SEM; *p < 0.05, **p < 0.01, ***p < 0.001 by *t*-test or Mann–Whitney *U*-test. **(G–K)** The relative expression of *mRNA Mcp1, Ccr2, Tnf*α*, Col1a, Il23a, Il22* of indicated genes in liver normalized to *Gapdh* (*n* = 6). The expression of *Mcp1, Ccr2, Tnf*α, and *Col1a* in Rag2^−/−^ mice was not different between Rag2^−/−^ mice fed with ND and HFD. However, in Rag2^−/−^ mice fed with HFD with the administration of A213 increased the expression **(G**–**J)**. The expression of *Il-23a* in liver in Rag2^−/−^mice fed with HFD was significantly higher than that in Rag2^−/−^mice fed with HFD fed with HFD (*p* = 0.009). **(K)** The administration of A213 further increased the relative expression of Il23a in both groups fed with ND and HFD (ND: *p* < 0.001, HFD: *p* = 0.010). **(L)** The administration of A213 further decreased the relative expression of *Il22* in both groups fed with ND and HFD (*p* < 0.001). Data are mean ± SEM; *p < 0.05, **p < 0.01, ***p < 0.001 by one-way ANOVA.

Flow cytometric analysis revealed that the number of CD45-positive cells in the liver per liver weight significantly increased by feeding with HFD (*p* = 0.037), and A213 administration significantly decreased the number of CD45-positive cells (Figure 5A). In addition, the ratio of ILC3s in CD45 positive cells and total ILCs in the liver significantly increased by feeding with HFD (*p* < 0.001), whereas A213 administration significantly decreased the ratio of ILC3s (Figures 5B,C). We also investigated the changes of M1/M2 macrophages in the liver (Supplementary Figure 2). The number of M1 macrophages in Rag2^−/−^ mice fed with HFD was significantly higher than that in Rag2^−/−^ mice fed with ND (*p* = 0.023) (Figure 5D), whereas the number of M2 macrophages in the liver of Rag2^−/−^ mice fed with HFD was significantly lower than that in Rag2^−/−^ mice fed with ND (*p* = 0.019) (Figure 5E). Moreover, the M1/M2 macrophage ratio in Rag2^−/−^ mice fed with HFD was significantly higher than that in Rag2^−/−^ mice fed with ND (*p* = 0.002) (Figure 5F). By the administration of A213, M1 macrophages increased and M2 macrophages decreased in both groups fed with ND and HFD.

The expression of *Mcp1, Ccr2, Tnfa*, and *Col1a* in Rag2^−/−^ mice fed with HFD and A213 administration significantly increased (*p* < 0.001). Moreover, the expression of *Il23a* in Rag2^−/−^ mice fed with HFD significantly increased (*p* = 0.034), and A213 administration increased the relative expression of *Il23a* in both group fed with ND and HFD (ND: *p* < 0.001, HFD: *p* = 0.021) (Figures 5G–K). Additionally, the administration of A213 further decreased the relative expression of *Il22* in both groups fed with ND and HFD (*p* < 0.001).

**Figure 6:**
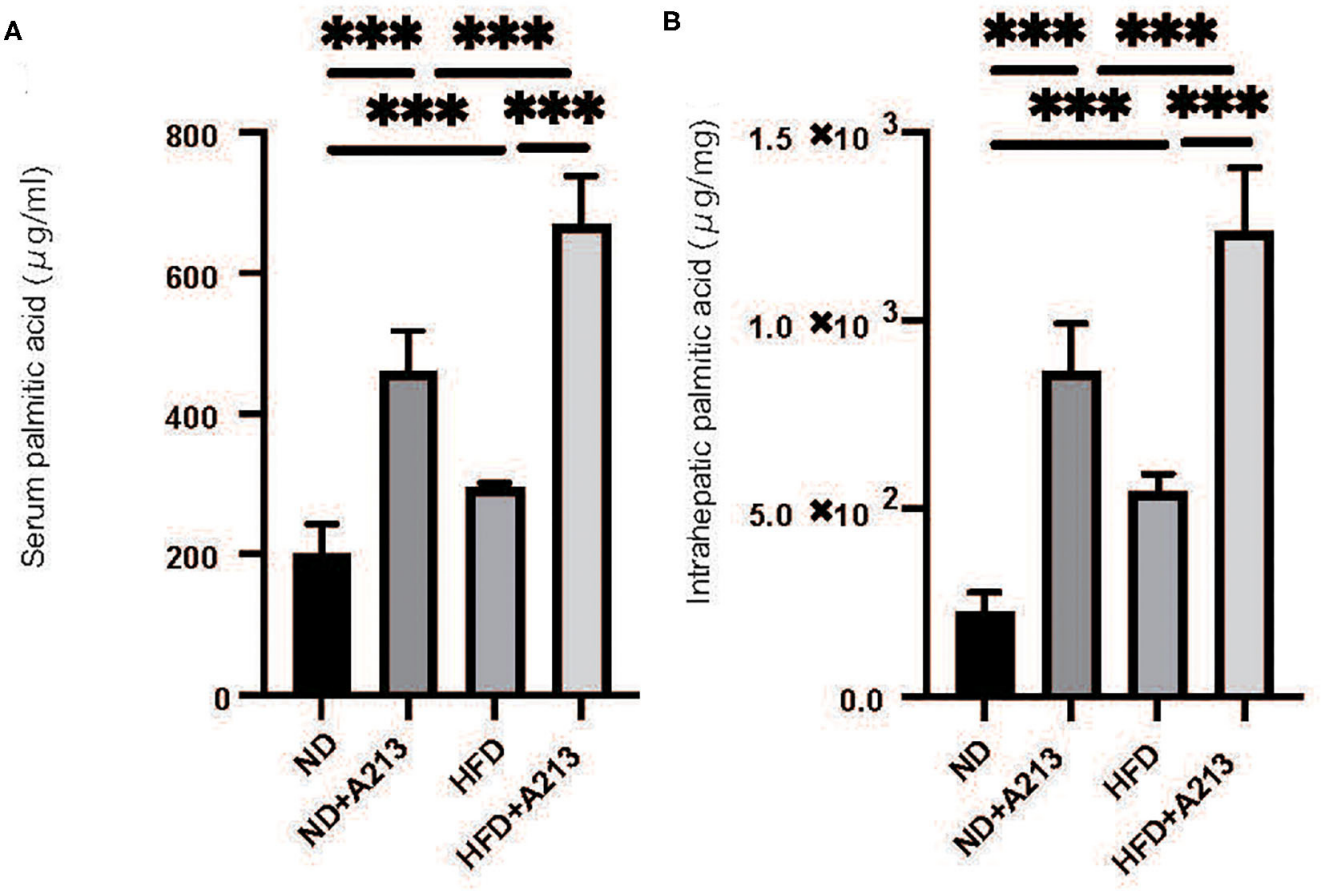
The administration of A213 caused increasing of palmitic acid. **(A)** Serum palmitic acid (μg/ml). **(B)** Intrahepatic palmitic acid (μg/mg). Serum and intrahepatic palmitic acids of Rag2^−/−^ mice with the administration of A213 were significantly higher than those without in both groups fed with ND and HFD (*p* < 0.001).

Serum palmitic acid levels were significantly higher in both ND and HFD groups with the administration of A213 (*p* < 0.001). In the liver, palmitic acid levels were significantly higher in both ND and HFD groups with the administration of A213 (Figures 6A–D).

### Palmitic Acid Induced Apoptosis of Primary Hepatocytes

**Figure 7:**
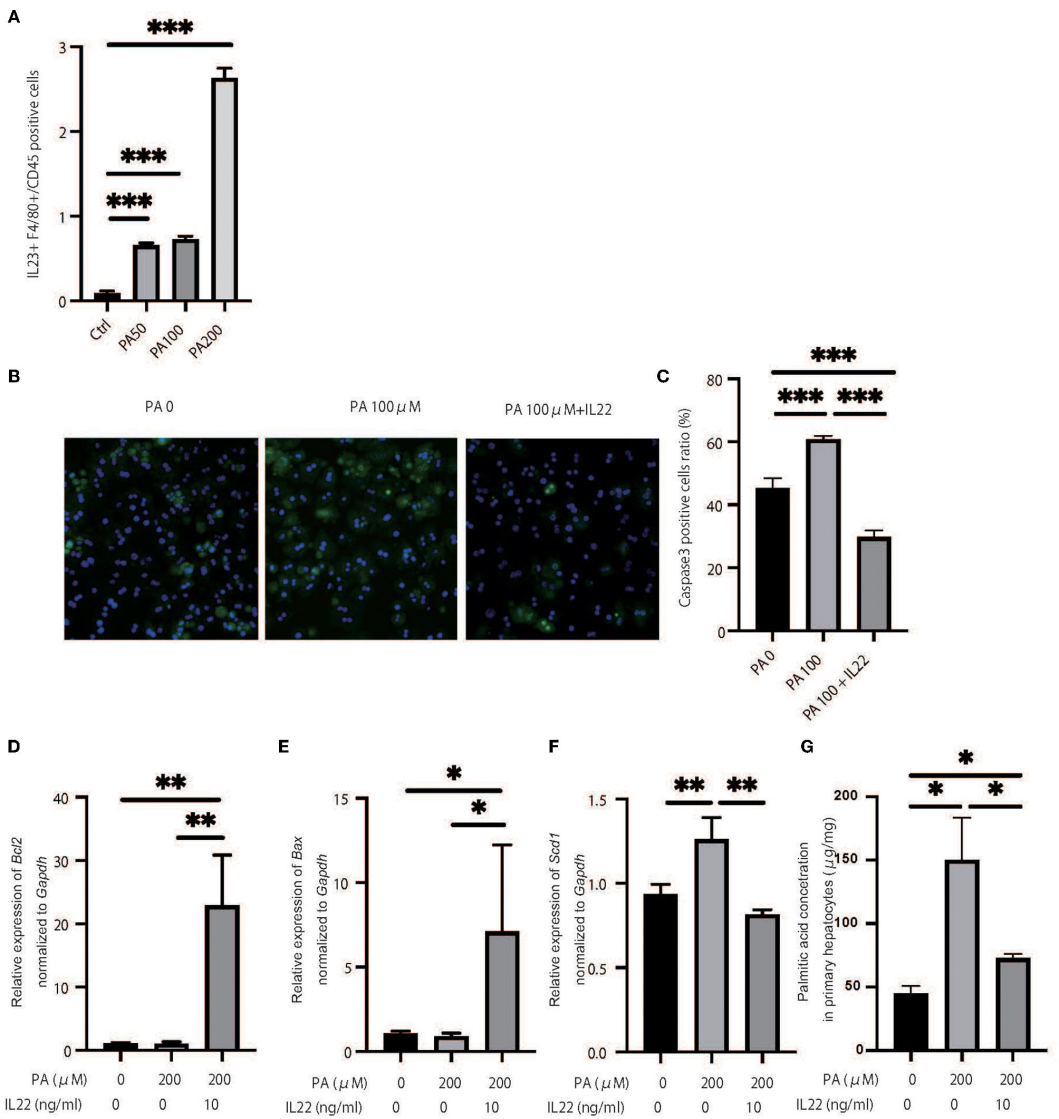
Palmitic acid induced secretion of IL-23 and palmitic acid with IL-22 induced anti-inflammatory effect. **(A)** The ratio of il-23+F4/80 RAW264.7 in CD45 positive cells. Treated with 100% ethanol (Ctrl), 50, 100, and 200 μM of palmitic acid (PA50, 100, 200). **(B)** Representative immunostaining of primary hepatocytes with Caspase3. Treated with 100% ethanol (PA 0), 100 μM of palmitic acid (PA 100), or 100 μM of palmitic acid and il22 (PA 100 + il-22). **(C)** The ratio of Caspase3 positive cells in an image (*n* = 6). The ratio of Caspase3 positive cells in primary hepatocytes in PA 100 was significantly higher than those in PA 0 and PA 100 + il-22 (PA0: *p* = 0.008, PA 100 + il-22: *p* = 0.002). **(D–F)** The relative expression of *mRNA bcl2, bax* and *scd1* in liver normalized to *Gapdh* (*n* = 6). Treated with 100% ethanol, 200 μM of PA, 200 μM of PA, and 200 μM of PA and 10 μg/ml of il-22. **(G)** The concentration of palmitic acid in primary hepatocytes (*n* = 6). The concentration of palmitic acid in primary hepatocytes treated with PA 100 + il-22 was significantly lower than that with PA (*p* = 0.014).

We hypothesized that a decrease in ILC3s caused an increase in palmitic acid in the liver and peripheral blood of RORgt KI/KI mice and Rag2^−/−^ mice with the administration of A213, and accumulation of palmitic acid caused inflammation and fibrosis in the liver. First, a murine macrophage-like cell line, RAW264.7, was used to evaluate the ability of fatty acids to secrete cytokines from macrophages. The addition of palmitic acids significantly increased the ratio of il-23^+^F480^+^ cells in CD45^+^ cells in relation to density (Figure 7A). Next, freshly isolated primary hepatocytes were plated on collagen-coated plates and treated with 100% ethanol (PA 0), 100 μM of palmitic acid (PA 100), or 100 μM of palmitic acid and il-22 (PA 100 + il-22). The ratio of Caspase 3-positive cells in primary hepatocytes was increased in PA 100, compared with PA 0, but that was decreased further by adding il-22 (PA0: *p* = 0.008, PA 100 + il-22: *p* = 0.002) (PA0: *p* = 0.008, PA 100 + il-22: *p* = 0.002) (Figures 7B,C). In addition the relative expression of *bcl2* and *bax*, which are genes related to anti-apoptosis, was significantly higher in hepatocytes treated with PA + il-22, compared to those treated with PA alone (Figures 7D,E). The expression of *scd1*, which is a key enzyme involved in the biosynthesis of monounsaturated fatty acids, was suppressed in hepatocytes treated with PA + il-22 compared to those treated with PA alone (Figure 7F). Finally, the concentration of palmitic acid in primary hepatocytes treated with PA I il-22 was significantly lower than that with PA alone (Figure 7G).

## Discussion

Our principal findings were that HFD induces an increase in ILC3s in the liver, and that the deficiency of ILC3s due to genetic modification or drug-induced leads to progression of liver fibrosis. In particular, excessive accumulation of palmitic acid in HFD without ILC3 was remarkable. It is widely known that liver damage due to long-term intake of HFD is caused mainly by saturated fatty acids (24, 25). Among all saturated fatty acids, palmitic acid has a pivotal role for liver damage. Immune defense mechanisms against the toxicity of saturated fatty acids exist in the body, and our study could demonstrate that ILC3s play a key protective role against the accumulation of palmitic acid (Graphical Abstract).

RORγt^gfp/gfp^ mice and Rag2^−/−^mice with the administration of A213, which impair the function of ILC3s, showed both significant liver fat accumulation and fibrosis. This means that ILC3s suppress chronic inflammation due to diet-induced obesity and consequently suppress liver fibrosis. In fact, feeding with HFD alone did not increase the gene expressions related to inflammation and fibrosis in the liver such as those of *Mcp1, Ccr2, Tnf*α, and *Col1a*. On the other hand, feeding with HFD and a deficiency of ILC3s, accompanied by a deficiency of acquired immunity, significantly increased the gene expression related to inflammation and fibrosis. Previous studies have reported the pivotal role of chronic inflammation in the pathogenesis of metabolic syndrome, obesity, and diabetes (26, 27). The prolonged low-level inflammatory responses due to chronic inflammation in metabolic syndrome impair tissue function and cause irreversible organ dysfunction by tissue remodeling, such as fibrosis (28). In the liver, chronic inflammation causes NAFLD and NASH (29). Recently, the role of innate immune cells, such as ILCs, in chronic inflammation has been reported by several groups (29, 30). ILCs do not express antigen-specific receptors; they respond to cytokines produced by surrounding macrophages, dendritic cells, and epithelial cells (9). Several studies revealed that ILCs regulate metabolism and obesity (31, 32). Wang et al. (20) demonstrated that ILC3s play a pro-fibrotic role in liver fibrosis progression. Moreover, Matsumoto et al. (13) reported that RORγt^−/−^ mice developed significant severe CCl4-induced hepatitis, compared to Rag2^−/−^ mice, and that ILC3s play a protective role in hepatitis. In our study, ILC3s play a protective role in the pathogenesis of NAFLD, because the deficiency of ILC3s led to significant deterioration of NAFLD, and it was speculated that ILC3 increased in the liver to prevent the progress of NAFLD. In line with Matsumoto's report, we also observed that Rag2^−/−^ mice, which lack acquired immunity, fed with HFD accumulated fat, but did not endure fibrosis in the liver.

In our lipidomics analysis, palmitic acid in both blood and liver in RORgt KI/KI mice was significantly elevated, compared to that in RORgt KI/w mice. Interestingly, deficiency of ILC3 caused significant liver fibrosis even in the absence of HFD administration. This was thought to be due to a significant increase in serum and hepatic palmitate levels due to ILC3 deficiency even within the group fed with ND. The following is a mechanistic discussion of how deficiency of ILC3 increased palmitic acid accumulation in liver tissue. As shown in the present study, deficiency of ILC3 increased M1 macrophages in the liver. IL-1β, secreted by M1 macrophages, promotes the translocation of CD36 (33), a fatty acid transporter, which increases the influx of long-chain fatty acids, including palmitic acid, into the hepatocytes. Lipids are digested by pancreatic lipase into fatty acids and glycerin. Glycerin is converted to dihydroxyacetone phosphate via glycerol-3-phosphate and metabolized through the glycolytic pathway. On the other hand, fatty acids are transported to the mitochondria and then metabolized to acetyl-CoA by β-oxidation. Mice displaying genetic ablation of the IL-22 receptor gene were prone to developing HFD-induced obesity and insulin resistance (34). In addition to that, long-term treatment with IL-22 decreased the hepatic expression of enzymes for lipid synthesis like ATP citrate lyase as well as elongation of very long chain fatty acids and reduced hepatic triglyceride and cholesterol levels (35). In this study, the administration of IL-22 decreased the concentration of palmitic acid in primary hepatocytes treated with palimitic acid. In Rag2KO and ROR KI/w mice, adipogenesis was clearly observed, but inflammatory fibrosis was mild. This might be due to the anti-inflammatory effect of IL22 induction by HFD. On the other hand, significant fibrosis was observed in ROR KI/KI mice, in which IL22 expression was reduced due to lack of ILC3, and in Rag2KO mice treated with A213. The reason why ROR KI/w mice or Rag2KO mice fed with HFD developed NAFLD but not NASH was that the ILC3 activated by HFD secreted IL22, and the effect of IL22 was thought to prevent hepatitis. Some previous studies demonstrated that palmitic acid is associated with pathogenesis of NAFLD in animal and human (24, 25). When palmitic acid binds to the toll-like receptor-4 of macrophages, the macrophages secrete cytokines, such as IL-12 and IL-23 and trigger an inflammatory response (36). In this study, the addition of palmitic acids into RAW264.7 significantly increased IL-23 positive cells. Furthermore, the addition of palmitic acids into primary hepatocytes induced significant apoptosis, whereas the further addition of IL-22 suppressed apoptosis and increased the expression of genes related to anti-apoptosis. Regarding the trends in intrahepatic macrophages in animal study, feeding with HFD significantly increases M1 macrophages and decreases M2 macrophages. M1 macrophages secrete IL-23 (37), which stimulates the production of IL-22 from ILC3s (23). Moreover, the addition of palmitic acid to primary hepatocytes increased the expression of scd1. On the other hand, the combination of palmitic acid and IL-22 decreased its expression. A high-fat-diet, and especially fatty acids such as palimitc acid, stimulates the production of IL-23 from M1 macrophages, which promotes ILC3s differentation. IL-22 secreted by ILC3s contributes to the upregulation of hepatic lipid metabolism and has anti-apoptotic activity, suggesting that ILC3 is increased in the liver in a compensatory manner to ameliorate liver damage caused by a high-fat diet.

## Summary

Innate lymphoid cells (ILCs) have been reported to play a key role in regulating metabolic disorders. The aim of our study was to investigate the role of group 3 ILCs (ILC3s) in the modulation of Non-alcoholic fatty liver disease (NAFLD). Our study reveals that ILC3s contribute to the upregulation of hepatic lipid metabolism and has anti-apoptosis activity.

## Data Availability Statement

The original contributions presented in the study are included in the article/Supplementary Material, further inquiries can be directed to the corresponding author/s.

## Ethics Statement

The animal study was reviewed and approved by The Committee for Animal Research at the Kyoto Prefectural University of Medicine.

## Author Contributions

MH originated and designed the study, researched the data, and wrote the manuscript. TO, TF, and YH originated and designed the study, researched the data, and reviewed the manuscript. KN, YY, SM, EU, NN, MA, and MY researched the data and contributed to the discussion. HT provided technical cooperation. MF originated and designed the study, researched the data, and reviewed and edited the manuscript. MH was the guarantor of this work and, as such, had full access to all of the data in the study and takes responsibility for the integrity of the data and the accuracy of the data analysis. All authors were involved in the writing of the manuscript and approved the final version of this article.

## Conflict of Interest

HT was employed by Agilent Technologies. The remaining authors declare that the research was conducted in the absence of any commercial or financial relationships that could be construed as a potential conflict of interest.
