# Supplementary material for: Group 3 Innate Lymphoid Cells Protect Steatohepatitis From High-Fat Diet Induced Toxicity
Source: Front Immunol. 2021 Mar 15;12:648754. doi: 10.3389/fimmu.2021.648754 (PMC8005651; doi:10.3389/fimmu.2021.648754)
Supplement: Supplementary file 1 [file Data_Sheet_1.PDF]

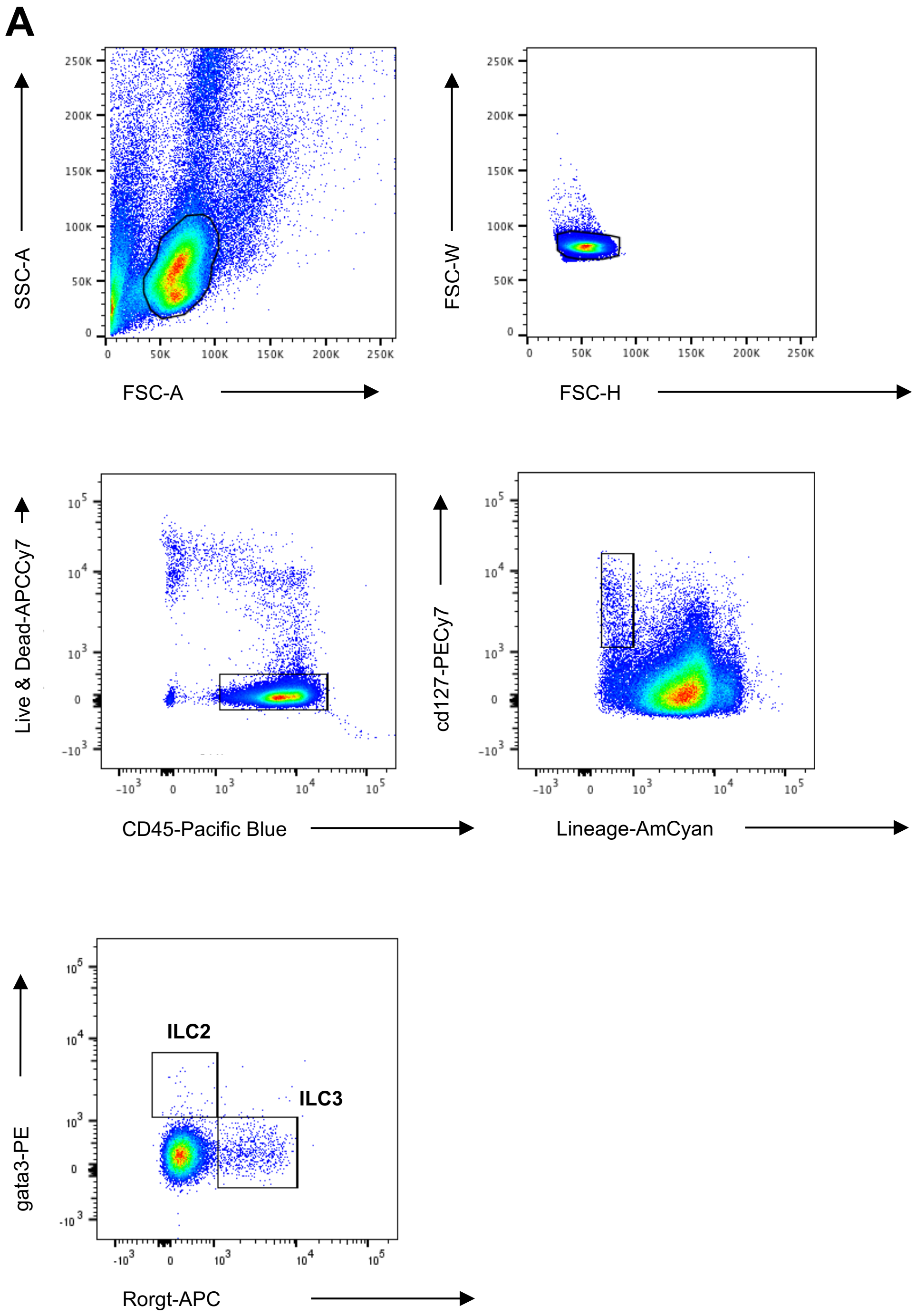

Supplementary Figure 1. Representative flow cytometry plots of liver CD45+ Live & Dead- Lin- CD127+ RORg- GATA-3+ ILC2s and CD45+ Live & Dead- Lin- CD127+ RORg+ GATA-3- ILC3s in each group.

Supplemental Figure 1

A

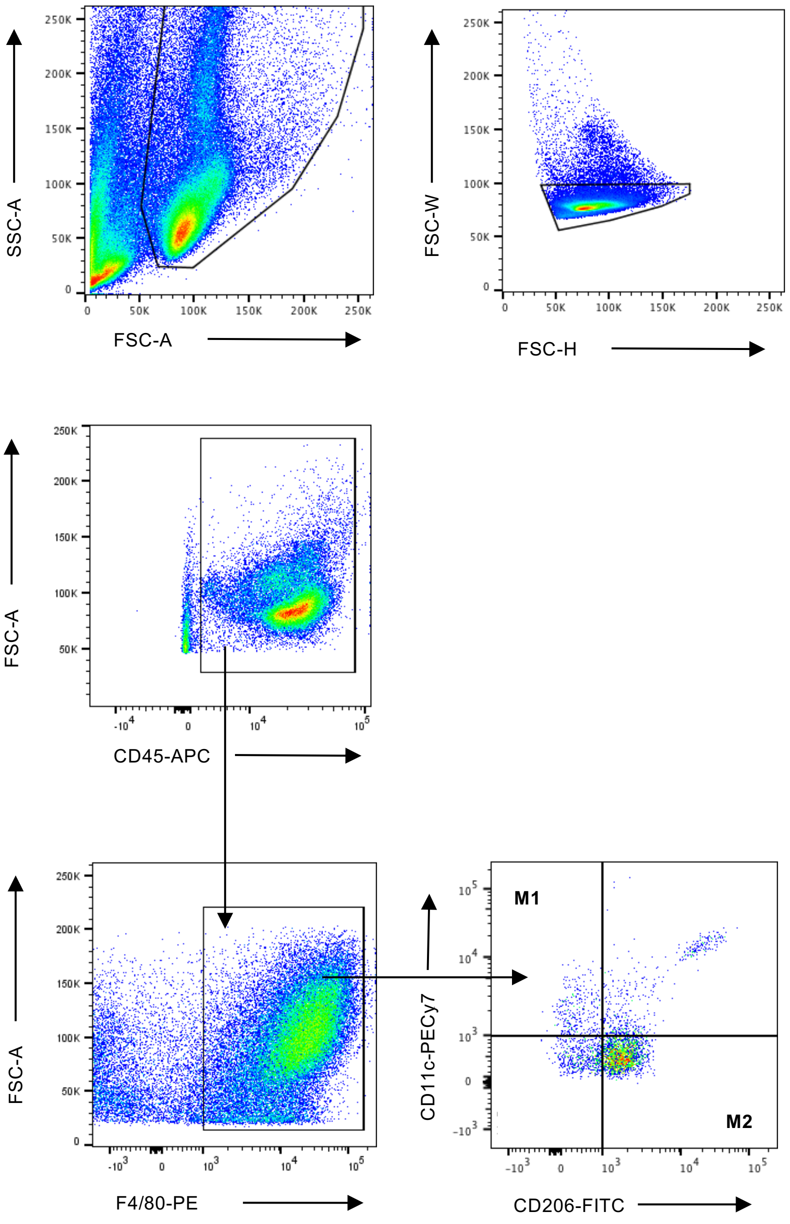

Supplementary Figure 2. Representative flow cytometry plots of liver CD45+ F4/80+ CD206- CD11c+ M1 macrophage and CD45+ F4/80+ CD206+ CD11c- M2 macrophage.

Supplemental Figure 2
